# Comparison of *K-ras* mutations in lung, colorectal and gastric cancer

**DOI:** 10.3892/ol.2014.2205

**Published:** 2014-05-30

**Authors:** NANQIU PENG, XINTAI ZHAO

**Affiliations:** Technical Department, Shanghai Shines Pharmaceuticals Company Limited, Research Base of Molecular Diagnosis Technology for Tumor Personalized Therapy, Development Center for Medical Science and Technology, Ministry of Health, Shanghai 201900, P.R. China

**Keywords:** *K-ras* mutation, lung cancer, colorectal cancer, gastric cancer, COLD-PCR

## Abstract

*K-ras* is involved in the EGFR pathway that regulates cell survival, motility and proliferation, as well as angiogenesis and metastasis. It is also essential for carcinogenesis. The *K-ras* mutation status can be used to predict the therapeutic efficacy of targeted drugs such as cetuximab. The aim of this study was to compare *K-ras* mutation in different types of cancer. Nested and COLD-PCR were used to detect *K-ras* mutations. The Chi-squared test was used for statistical analysis. In this study, the total *K-ras* mutation frequency was found to be 9.09, 18.61 and 6.67% in lung, colorectal and gastric cancer, respectively. Similar *K-ras* mutation frequencies were detected among sample types and genders for lung and gastric cancer, with the exception of colorectal cancer. However, age had no impact on the *K-ras* mutation rates.

## Introduction

Cancer is a global disease with a high incidence of mortality, having caused 7.6 million mortalites in 2008 alone. Over the past 10 years, there has been a decreasing trend in mortality due to cancer, allowing for the prevention of ~1.18 million cancer-related mortalities (1,2).

Lung, colorectal and gastric cancer are the leading cancer types in terms of occurrence and severity: lung cancer is the most common cancer worldwide and the first leading cause of cancer mortality, colorectal cancer is the third most common cancer worldwide and the fourth leading cause of cancer-related mortality, while gastric cancer is the fourth most common cancer worldwide and the third leading cause of cancer-related mortality (1). The incidence of cancer has seen a steady decline in males while it has remained stable in females, resulting in a gradual decrease in the overall mortality rate with regard to cancer (2). Thus NCCN guidelines suggest that the *K-ras* mutation be detected prior to applying medication such as cetuximab.

## Materials and methods

### Patients

Clinical samples were obtained from 100 hospitals in China, including 131 tissue samples, 51 plasma samples, and 5 pleural and ascites samples for lung cancer; 445 tissue and 60 plasma samples for colorectal cancer; and 126 tissue and 9 plasma samples for gastric cancer. Approval for this study was obtained from the Shanghai Clinical Research Center Ethics Committee. All patients participating in this study provided written informed consent.

Tissue samples were stored and transported under controlled temperatures, while plasma, and pleural and ascites samples were transported on ice packs. The following materials were purchased: Taq DNA polymerase (Takara Biotechnology Co., Ltd., Dalian, China), dNTP (Shi Ze Biotechnology Co., Ltd., Shanghai, China), the DNA extraction kit (DN10, Aidlab Biotechnologies Co., Ltd., Beijing, China), PCR instrument (EDC-810, Eastwin Biotechnology Co., Ltd., Beijing, China), BioSafe Centrifuge Systems (L420, Xiangyi LXJ Centrifuge Instruments Co., Ltd.).

### Methods

Nested and COLD-PCR were used to detect the *K-ras* mutations. Regular PCR was used to amplify the 465-bp outer product. The primers used were: forward, 5′-GTCGATGGAGG AGTTTGTAAATGAAGT-3′ and reverse 5′-TTCAGATAACTTAACTTTCAGCATAATTATCTTG-3′. This was followed by 10 μl PCR reaction mixture including 0.25 mM dNTP, 0.5 μM primers, 0.5 units Taq DNA polymerase and 10 ng DNA template. The PCR program was conducted under the following conditions: 3 min at 95°C for 1 cycle, 32 amplification cycles for 30 sec at 94°C, 30 sec in 57°C, and 30 sec at 72°C, and maintained for 5 min at 72°C. COLD-PCR was used to amplify the 155-bp inner product. The primers used were: forward, 5′-GTCACATTTT CATTATTTTTATTATAAGG-3′ and reverse 5′-TTTACCTCTATTGTTGGATCATATTC-3′. This was followed by 50 μl PCR reaction mixture including 0.25 mM dNTP, 0.5 μM primers, 0.5 units Taq DNA polymerase and 1 μl outer PCR product. The PCR program was conducted under the following conditions: 3 min at 95°C for 1 cycle, 40 amplification cycles for 30 sec at 80°C, 30 sec in 58°C, 30 sec at 72°C, followed by 15 cycles for 30 sec at 94°C, 30 sec at 58°C, and 30 sec at 72°C, maintained for 5 min at 72°C.

The Chi-squared test was used for statistical analysis. P<0.05 was considered statistically significant.

## Results

**Figure 1:**
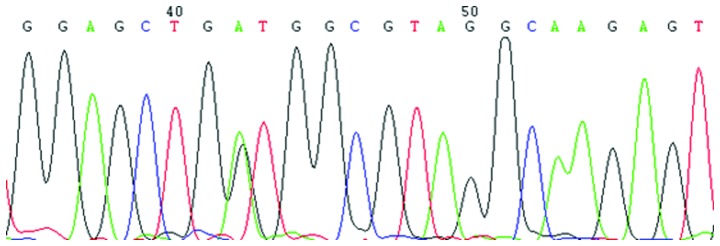
Representative sequencing chromatogram of COLD-PCR product, showing the GGT mutation to GAT at codon 12 of *K-ras* gene in a colorectal cancer patient.

The *K-ras* mutation frequency was detected in lung cancer (Table I), colorectal cancer (Table II) and gastric cancer (Table III). COLD-PCR was used to detect the *K-ras* mutations. Fig. 1 shows the representative results, which showed the G12D (GGT>GAT) mutation.

The total *K-ras* mutation frequency was 9.09, 18.61 and 6.67% in lung, colorectal and gastric cancer, respectively, as detected in all types of samples which suggested that the *K-ras* mutations occurred more frequently in colorectal cancer than in the other two types of cancer investigated.

Of 187 lung cancer patients investigated, four mutation types were detected, including G12C (GGT>TGT), G12D (GGT>GAT), G12V (GGT>GTT) and G13D (GGC>GAC). The mutation frequency was 1.96, 11.45 and 20% in plasma, tumor tissue, and pleural and ascites samples, respectively, with no statistical significance being identified (P=0.0935). The ratio of male to female patients was 8.94 and 9.38%, respectively, which did not indicate statistical significance (P=0.9223). Similarly, the ratio for youth, middle age, and elderly patients was 11.11, 6.94 and 10.38%, respectively, which did not indicate statistical significance (P=0.7196).

Of 505 colorectal cancer patients investigated, 11 mutation types were detected, including G12S (GGT>AGT), G12R (GGT>CGT), G12C (GGT>TGT), G12D (GGT>GAT), G12A (GGT>GCT), G12V (GGT>GTT), G13S (GGC>AGC), G13R (GGC>CGC), G13C (GGC>TGC), G13D (GGC>GAC) and Q61L (CAA>CTA). The mutation frequency for plasma, and tumor tissue samples was 5 and 20.45%, respectively, indicating statistical significance (P=0.0039). The ratio for male to female patients was 14.97 and 23.70%, respectively, indicating statistical significance (P=0.0129). Similarly, the ratio for youth, middle age, and elderly patients was 8.33, 21.03 and 19.2%, respectively, which did not indicate statistical significance (P=0.0824).

Of 135 gastric cancer patients investigated, the mutation types G12D (GGT>GAT), and G13D (GGC>GAC) were detected. The mutation frequency for plasma, and tumor tissue samples was 0 and 7.14%, respectively, which did not indicate statistical significance (P=1.0000). The ratio for male to female patients was 7.61 and 4.65%, respectively, which indicated no statistical significance (P=0.7860). Similarly, the ratio for youth, middle age, to elderly patients was 9.09, 4.92 and 7.69%, respectively, which indicated no statistical significance (P=0.7425).

Of all the *K-ras* mutation types in lung cancer, G12C accounted for 1.07%, G12D for 3.21%, G12V for 2.14%, G13D for 2.67% of the total mutation frequency. Analysis of colorectal cancer mutations showed that, G12D accounted for 7.13%, G12V for 2.97%, G13D for 4.95%, while the remaining mutation types collectively accounted for 3.56% of the total mutation frequency (Table II). In gastric cancer patients, only two *K-ras* mutation types were identified, with G12D accounting for 2.22%, whereas G13D accounted for 4.44% of the total mutation frequency. Thus, G12D and G13D are the two most frequently occurring mutation types in the three types of cancer investigated.

## Discussion

The mammalian *ras* gene family comprises *H-ras*, *K-ras*, *N-ras*, encoding H-ras, K-ras, N-ras proteins, respectively, with a similar structure and function. The Ras protein is located in the inner region of the cell membrane, tranforms signals from EGFR to mitogen-activated protein kinases (MAPKs), to control cell growth, proliferation, and motility, as well as metastasis and angiogenesis (3). The *K-ras* gene usually contains point mutations at codons 12, 13 and 61 (Tables I–III), and these mutations often activate the *K-ras* oncogene (4,5). The *K-ras* mutation status is associated with the therapeutic efficacy of EGFR-targeting monoclonal antibodies, rendering patients with *K-ras* mutation as not suitable for Erbitux treatment (6).

Various methods have been developed to improve detection sensitivity, such as denaturing high-performance liquid chromatography (DHPLC) (7), nested Allele-Specific Blocker (ASB-)PCR (8), PCR single-strand conformation polymorphism (SSCP) (9), restriction fragment length polymorphism (RFLP) (10), and the amplification refractory mutation system (ARMS) (11). Due to the need for simple equipment, high sensitivity, COLD-PCR (co-amplification at lower denaturation temperature-PCR) (12–15) has been widely used, it can enrich variant DNA sequences and improve detection sensitivity.

In the present study, the results showed that the mutation frequency of *K-ras* was different in the three types of cancer, indicating statistical significance (P=0.0001). The ratio for the variables compared was highest in colorectal cancer. Thus, detection of *K-ras* mutation status is more important for colorectal cancer patients when personalized medicine is involved.

The mutation frequency was not statistically significant for the different sample types for lung and gastric cancer. Therefore, plasma samples may be substituted by tissue samples when the latter are not readily available, particularly for lung cancer patients, from whom pleural and ascites samples are also feasible. However, other types of samples cannot be substituted for colorectal cancer tissues for *K-ras* mutation detection, considering that the detection frequency of *K-ras* mutations in tumor tissues is 4-fold that of plasma samples, with the difference between sample types being statistically significant for colorectal cancer patients.

For lung cancer and gastric cancer patients, the mutation frequency indicated no statistical significance for gender, although a difference was identified for colorectal cancer. The frequency for male to female was 14.97 and 23.70% (P=0.0129), respectively, suggesting the likelihood of mutation in female colorectal cancer patients as compared with their male counterparts.

Age did not affect the mutation frequency in the three types of cancer investigated, suggesting that *K-ras* mutation does not play a role in patient age Previously, an anticorrelation pattern of *K-ras* mutation status with the therapeutic effect, progression-free survival and overall survival following patient treatment with Erbitux was demonstrated (4,6). By contrast, results of other studies have shown that many patients cannot improve efficacy end-points after receiving Erbitux (16,17) without *K-ras* mutation detection. That finidng suggests that other key signal transduction molecules also play an important role in the downstream of Erbitux against EGFR, for example, B-raf, PIK3CA (17). Therefore, the mutation status of genes such as *B-raf*, and *PIK3CA* should be detected at the same time as the *K-ras* mutation status.

## Figures and Tables

**Table I:** The mutation frequencies of *K-ras* gene in different sample types, genders and age groups of lung cancer patients detected with COLD-PCR and sequencing.

		Mutation frequency (%)							
		
		Type of samples			Gender		Age (years)		
				
Amino acid change	*K-ras* mutation	Plasma N=51	Tumor tissue N=131	Pleural and ascites N=5	Male N=123	Female N=64	Youth (25–44) N=9	Middle age (45–59) N=72	Elderly (60–90) N=106
G12S	GGT>AGT								
G12R	GGT>CGT								
G12C	GGT>TGT		2 (1.53)		2 (1.63)			1 (1.39)	1 (0.94)
G12D	GGT>GAT		5 (3.82)	1 (20)	4 (3.25)	2 (3.13)		1 (1.39)	5 (4.72)
G12A	GGT>GCT								
G12V	GGT>GTT		4 (3.05)		2 (1.63)	2 (3.13)		1 (1.39)	3 (2.83)
G13S	GGC>AGC								
G13R	GGC>CGC								
G13C	GGC>TGC								
G13D	GGC>GAC	1 (1.96)	4 (3.05)		3 (2.44)	2 (3.13)	1 (11.11)	2 (2.78)	2 (1.89)
G13A	GGC>GCC								
G13V	GGC>GTC								
Q61K	CAA>AAA								
Q61L	CAA>CTA								
Q61H	CAA>CAT								
Total (%)		1.96	11.45	20	8.94	9.38	11.11	6.94	10.38

**Table II:** The mutation frequencies of *K-ras* gene in different sample types, genders, and age groups of colorectal cancer patients detected with COLD-PCR and sequencing.

		Mutation frequency							
		
		Type of samples			Gender		Age (years)		
				
Amino acid change	*K-ras* mutation	Plasma N=60	Tumor tissue N=445	Pleural and ascites N=0	Male N=294	Female N=211	Youth (25–44) N=60	Middle age (45–59) N=195	Elderly (60–90) N=250
G12S	GGT>AGT		2 (0.45)			2 (0.95)		1 (0.51)	1 (0.4)
G12R	GGT>CGT		1 (0.22)		1 (0.34)				1 (0.4)
G12C	GGT>TGT		5 (1.12)		3 (1.02)	2 (0.95)		1 (0.51)	4 (1.6)
G12D	GGT>GAT	1 (1.67)	35 (7.87)		14 (4.76)	22 (10.43)	3 (5)	12 (6.15)	21 (8.4)
G12A	GGT>GCT		4 (0.90)		3 (1.02)	1 (0.47)		2 (1.03)	2 (0.8)
G12V	GGT>GTT	1 (1.67)	14 (3.15)		9 (3.06)	6 (2.84)		8 (4.10)	7 (2.8)
G13S	GGC>AGC	2 (0.45)				2 (0.95)			2 (0.8)
G13R	GGC>CGC		1 (0.22)		1 (0.34)			1 (0.51)	
G13C	GGC>TGC		1 (0.22)		1 (0.34)				1 (0.4)
G13D	GGC>GAC	1 (1.67)	24 (5.39)		11 (3.74)	14 (6.64)	2 (3.33)	15 (7.69)	8 (3.2)
G13A	GGC>GCC								
G13V	GGC>GTC								
Q61K	CAA>AAA								
Q61L	CAA>CTA		2 (0.45)		1 (0.34)	1 (0.47)		1 (0.51)	1 (0.4)
Q61H	CAA>CAT								
Total (%)		5	20.45		14.97	23.70	8.33	21.03	19.2

**Table III:** The mutation frequencies of *K-ras* gene in different sample types, genders, and age groups of gastric cancer patients detected with COLD-PCR and sequencing.

		Mutation frequency							
		
		Type of samples			Gender		Age (years)		
				
Amino acid change	*K-ras* mutation	Plasma N=9	Tumor tissue N=126	Pleural and ascites N=0	Male N=92	Female N=43	Youth (25–44) N=22	Middle age (45–59) N=61	Elderly (60–90) N=52
G12S	GGT>AGT								
G12R	GGT>CGT								
G12C	GGT>TGT								
G12D	GGT>GAT		3 (2.38)		3 (3.26)		1 (4.55)	1 (1.64)	1 (1.92)
G12A	GGT>GCT								
G12V	GGT>GTT								
G13S	GGC>AGC								
G13R	GGC>CGC								
G13C	GGC>TGC								
G13D	GGC>GAC		6 (4.76)		4 (4.35)	2(4.65)	1 (4.55)	2 (3.28)	3 (5.77)
G13A	GGC>GCC								
G13V	GGC>GTC								
Q61K	CAA>AAA								
Q61L	CAA>CTA								
Q61H	CAA>CAT								
Total (%)		0	7.14		7.61	4.65	9.09	4.92	7.69
